# Loss-of-heterozygosity on chromosome 19q in early-stage serous ovarian cancer is associated with recurrent disease

**DOI:** 10.1186/1471-2407-12-407

**Published:** 2012-09-12

**Authors:** Ingiridur Skirnisdottir, Markus Mayrhofer, Maria Rydåker, Helena Åkerud, Anders Isaksson

**Affiliations:** 1 Department of Women’s and Children’s Health, Uppsala University, SE-751 85, Uppsala, Sweden; 2 Science for Life Laboratory, Department of Medical Sciences, Uppsala University, SE-751 85, Uppsala, Sweden

**Keywords:** Allele-specific copy number, FFPE, LOH, Prognosis, Serous ovarian cancer, TAPS, Early-stage

## Abstract

**Background:**

Ovarian cancer is a heterogeneous disease and prognosis for apparently similar cases of ovarian cancer varies. Recurrence of the disease in early stage (FIGO-stages I-II) serous ovarian cancer results in survival that is comparable to those with recurrent advanced-stage disease. The aim of this study was to investigate if there are specific genomic aberrations that may explain recurrence and clinical outcome.

**Methods:**

Fifty-one women with early stage serous ovarian cancer were included in the study. DNA was extracted from formalin fixed samples containing tumor cells from ovarian tumors. Tumor samples from thirty-seven patients were analysed for allele-specific copy numbers using OncoScan single nucleotide polymorphism arrays from Affymetrix and the bioinformatic tool Tumor Aberration Prediction Suite. Genomic gains, losses, and loss-of-heterozygosity that associated with recurrent disease were identified.

**Results:**

The most significant differences (p < 0.01) in Loss-of-heterozygosity (LOH) were identified in two relatively small regions of chromosome 19; 8.0-8,8 Mbp (19 genes) and 51.5-53.0 Mbp (37 genes). Thus, 56 genes on chromosome 19 were potential candidate genes associated with clinical outcome. LOH at 19q (51-56 Mbp) was associated with shorter disease-free survival and was an independent prognostic factor for survival in a multivariate Cox regression analysis. In particular LOH on chromosome 19q (51-56 Mbp) was significantly (p < 0.01) associated with loss of TP53 function.

**Conclusions:**

The results of our study indicate that presence of two aberrations in TP53 on 17p and LOH on 19q in early stage serous ovarian cancer is associated with recurrent disease. Further studies related to the findings of chromosomes 17 and 19 are needed to elucidate the molecular mechanism behind the recurring genomic aberrations and the poor clinical outcome.

## Background

Epithelial ovarian cancer (EOC) is the leading cause of death among women with gynecologic malignancies [1]. In recent years it has become accepted that there are five histo-pathological subtypes of EOC (high-grade serous, mucinous, endometrioid, clear cell and low-grade serous), which represent five different diseases with different biological and genetic backgrounds. Common genetic alterations and biomarker expression is more strongly associated with histological sub-type than with stage of the disease [2-5] and as a consequence of that they should be analyzed separately [2,6].

Serous ovarian cancer is usually diagnosed at an advanced stage (FIGO-stage III-IV). Most cases of early-stage serous ovarian cancers are potential low malignant tumors which develop after stepwise progression from borderline tumors (Type I). This is in contrast to stage I high-grade serous ovarian cancer (Type II) which is a rare disease [7-9]. Recurrence of early stage (FIGO-stages I-II) ovarian cancer results in survival that is comparable to those with recurrent advanced-stage disease [1,10]. For apparently similar cases of ovarian cancer, the biological behaviour, response to treatment, and prognosis vary, and there is a need for molecular markers that identify which group of patients that are in need of adjuvant treatment and who are not [11,12].

Ovarian carcinomas are frequently afflicted by numerous deletions, amplifications and translocations throughout the genome. These genomic alterations may change the number of chromosomes (aneuploidy) and affect the average ploidy of the genome. Deletions and loss-of-heterozygosity (LOH) are of particular importance for tumor suppressor genes as they may have removed the last functional gene copy, allowing previously sustained or inherited recessive mutations to influence the tumor phenotype [13].

DNA copy number changes in specific genes in micro-dissected ovarian tumor tissue samples have been investigated previously by cDNA array analysis [14,15]. Several groups have used microarrays as a tool to predict outcome of patients in early [9] or advanced stages of ovarian cancer [5]. As chromosomal aberrations might reflect oncogene activation and loss of tumor suppressor genes, genome-wide analysis of copy number gain and loss is important in the search for predictive factors for chemoresistance and survival in ovarian cancer [13,15]. Array based comparative genomic hybridization (array CGH) and single nucleotide polymorphism (SNP) arrays are key techniques to analyze copy number alterations (CNA) that allow detection of allele-specific copy number and thereby LOH – even in tumor tissue affected by aneuploidy and a high proportion of normal cells [15,16].

In the clinics, biopsies of cancer resection specimens are fixed in formalin and embedded in paraffin (FFPE) for routine diagnostic histopathology. These biopsies on which the primary histo-pathological diagnosis are made, have not been affected by any kind of cancer therapeutics before collection and represent precious tissue samples for molecular microarray analysis [17].

In this hypothesis generating study we analyzed possible genomic alterations associated with recurrent disease in early-stage serous ovarian cancer compared with high-grade and low/medium grade serous ovarian tumors. We found that presence of two aberrations in TP53 on 17p and LOH on 19q in early stage serous ovarian cancer might be associated with recurrent disease.

## Methods

### Patients and tumor specimens

From January 2000 to December 2004, 51 patients with serous ovarian cancer in FIGO-stages I-II were recruited in the Uppsala-Örebro Medical region in Sweden after histological confirmation of epithelial ovarian carcinoma and presence of tumor cells in haematotoxylin-eosin stained slides. This study assessing 51 patients were a part of a patient population of 131 patients in earlier published studies [18-20]. All patients underwent primary surgery and postoperative chemotherapy. Adequate (optimal or modified) surgical staging according to the EORTC surgical staging categories in early ovarian cancer was undertaken in 7 (19%) out of the 37 cases, and in the remaining 30 (81%) patients surgical staging was regarded as inadequate according the same guidelines [21].

All samples were collected with the patient’s informed consent in compliance with the Helsinki Declaration [22], and used in accordance with the Swedish Biobank Legislation and Ethical Review Act (approval by Uppsala Ethical Review Board, decision ref.UPS-03-477). Pathology review was conducted from FFPE samples adjacent to the tissue from which DNA was extracted after manually micro-dissection with a scalpel to enrich for tumor cells. In addition, DNA-extraction from 11 normal samples from the contra-lateral ovary taken from matched paraffin block collections, were used to reduce the variability in the data. DNA was extracted as previously described [17,23]. In total, 37 out of 51 samples remained after quality control. The 51 samples had previously been analyzed with standard protocol for FFPE samples on 250K Affymetrix SNP arrays. With this method only ten of the samples produced data allowing copy number analysis (data not shown). In Table 1 summary of clinico-pathologic features of patients belonging to samples are presented.

**Table 1:** Patient characteristics (N = 37)

**Age (mean)**	**62.5 (range 45-84)**	
	**N**	**%**
FIGO-stage		
IA	7	(18.9)
IB	3	(8.1)
IC	21	(56.8)
II	6	(16.2)
FIGO-grade		
Grade 1	11	(29.7)
Grade 2	11	(29.7)
Grade 3	15	(40.6)
Status of the recurrent disease		
Recurrent disease	13	(35.1)
Without recurrent disease	24	(64.9)

### OncoScan

Genomic DNA was extracted from 51 FFPE Serous Ovarian cancer samples in compliance with the service provider's instructions for Affymetrix MIP_cn_330K/OncoScan™ FFPE Express Services and sent to Affymetrix Research Services Laboratory (ARSL) for processing [23]. Total and allele-specific SNP intensities received from ARSL were subjected to Rank Segmentation Nexus Copy Number 4.0, a slightly modified Circular Binary Segmentation [24].

### Copy number analysis

Total and allele-specific intensities received from Affymetrix Research Services Laboratory (ARSL) were analyzed with Tumor Aberration Prediction Suite (TAPS) [16]. Originally designed and validated for Affymetrix SNP 6.0 and 500k arrays, no significant adjustments of TAPS were needed for analysis of OncoScan data.

### Statistical analysis

Fishers exact test (p < 0.05) identified differences in frequencies of total copy number for amplification, deletion and LOH in samples between the subgroups with and without recurrent disease. The Pearson's Chi-square test was used for testing proportional differences in univariate analyses. The survival curves were generated by using the Kaplan-Meier technique and differences between these curves were examined by the log-rank test. All tests were two-sided and the level of statistical significance was P ≤ 0.05. The Statistica 10 (StatSoft™) statistical package was used for the analyses. For multivariate analyses the logistic regression model was used with recurrence as the end point and Cox regression model was used with disease-free survival (DFS) as the endpoint.

## Results

### Background characteristics

Genomic DNA from fifty-one patients was extracted from pathologist-annotated FFPE ovarian tumors. The DNA was analyzed using high resolution (330K) OncoScan SNP arrays and subsequent allele-specific copy number analysis was carried out using the bioinformatic tool Tumor Aberration Prediction Suite (TAPS) [16]. The analysis failed for 14 samples mainly due to insufficient PCR product or low tumor cell content. The characteristics of the remaining ovarian cancer samples from 37 patients for which copy number information was obtained are described in Table 1. Since no differences with regard to age, FIGO-stage (p = 0.252), grade (p = 0.880), or recurrent disease (p = 0.657) could be detected between the 37 analyzed patients and the subgroup with failing samples, there was no evidence of the failing samples causing an ascertainment bias.

### Allele-specific copy number analysis

**Figure 1:**
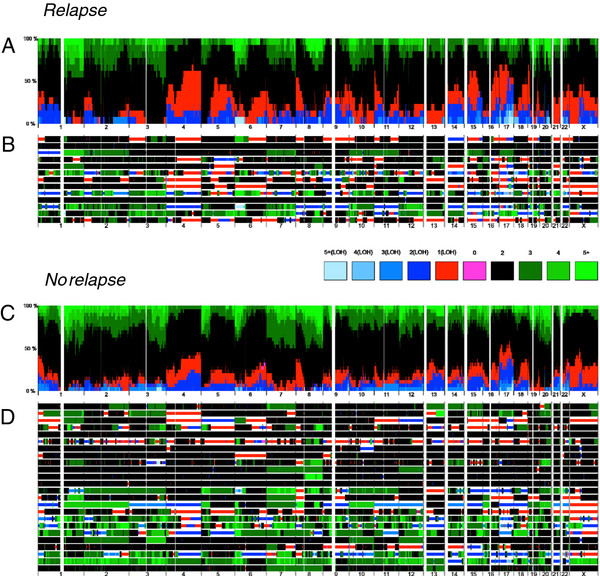
**Allele-specific copy number analysis in early stage serous ovarian cancers across the genome.** Frequency of aberrations with different total copy number and LOH in tumors with recurrent disease (**A**) and no recurrence (**C**). Individual total copy number and LOH for each tumor with recurrent disease (**B**) and no recurrence (**D**).

**Figure 2:**
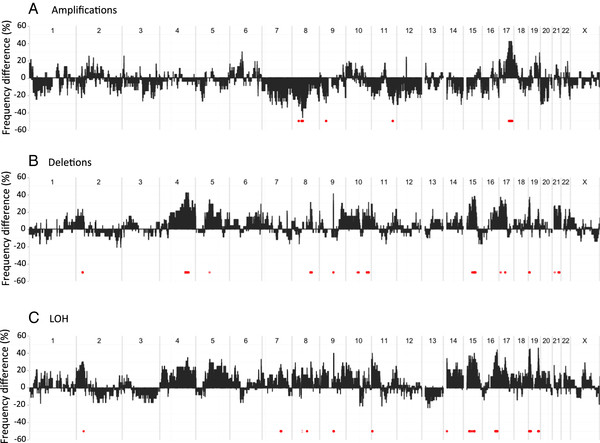
**Differences in frequency of tumors with aberrations between tumor with and without recurrence across the genome.** Gains (total copy number 3 and higher) (**A**). Total copy number 1 (**B**) and LOH irrespective of total copy number (**C**).

In 33 out of the 37 samples the allele-specific copy number analysis of the tumor genomes showed large aberrations affecting several chromosomes. The average copy number deviated significantly from two in several samples and only four samples showed minimal signs of copy number alterations. LOH was widespread, most of it in the form of loss to a single copy. No regions of recurrent homozygous loss or high-gain (six or more copies) were found. In Figure 1 the results of the copy number analysis are summarized as frequency of individual total copy number and LOH status in patients with (n = 13) and without (n = 24) recurrent disease. Individual frequencies of gain, loss and LOH in the patient groups are available in Additional file 1. Genomic regions where aberrations were associated with recurrent disease were identified by comparing the frequencies of gain (> 2 copies), loss (< 2 copies) and LOH (irrespective of total copy number) in the groups, using Fisher’s exact test (p < 0.05) to assess significance. Genome-wide differences in frequency are presented in Figure 2. Significantly different frequencies of gain were found on chromosomes 8p, 9p, 11q and 17q (Additional file 2). Similarly, significantly different frequencies of losses were found on chromosomes 2p, 4q, 5q, 8q, 9q, 10q 15q, 17p and q, 19p and 21q (Supplement 2). LOH was found to be common in many regions with frequencies of up to about 80% (Supplement 1).LOH was particularly frequent (up to about 80%) on chromosome 4q in both groups, implying presence of a mutated tumor suppressor gene. Significant differences in frequencies between the subgroups of patients with and without recurrent disease could be identified on chromosomes 2p, 7q, 8q, 9q, 11 p, 15q, 16q and 19p and q (Figure 2C). The regions with the largest difference in LOH frequency (≥ 36 percentage units) were located to chromosome 7, chromosome 16 and chromosome 19. In summary 519 genes were located in regions where LOH was significantly more frequent in samples with recurrence.

### LOH on chromosome 19 and association with loss of TP53 function

The most significant differences (p < 0.01) in LOH were seen in two relatively small regions of chromosome 19; 8.0-8,8 Mbp (19 genes) and 51.5-53.0 Mbp (37 genes). Thus, totally 56 genes on chromosome 19 were potential candidate genes associated with clinical outcome.

Recessive mutations in the TP53 gene is a well-known mechanism involved in tumor progression and a high frequency (up to 80%) of LOH around TP53 on 17p was observed. We used information on TP53 status from a previous immunohistochemistry study using the antibody DO-7 on a larger cohort including the samples in this study [18]. Loss of TP53 function (TP53-positivity) was found in 12/37 (32%) patients in this study (Table 2). We observed that loss of p53-function was significantly associated with LOH at 19q (51-56 Mbp) (p = 0.008). Thus, presence of two aberrations i.e. functional loss of TP53 on 17p and LOH on 19q in a tumor genome was associated with recurrence of the tumor.

**Table 2:** Clinical, pathological and genomic features vs recurrent disease (N = 37)

	**N% Recurrent disease (N = 13) (35%)**	**N% Non-recurrent disease (N = 24) (64.9%)**	**P value**
Age(mean)	64.0 years	61.8 years	p = 0.612 (t-test)
Tumor grade			
G1 + G2	8.(62)	14 (58)	
G3	5 (38)	10 (42)	p = 0.849 (chi-2)
FIGO stage			
IA-IC	8 (61)	23 (96)	
II	5 (42)	1 (4)	p = 0.007 (chi-2)
Staging			
adequate	1 (8)	6 (25)	
not adequate	12 (92)	18 (75)	p = 0.199 (chi-2)
LOH detected			
at19q (51-56Mbp)	8 (62)	3 (12)	
No. LOH at 19q	5 (38)	21 (88)	p = 0.002 (chi 2)
(51-56Mbp) detected			
TP53 status			
positive	8 (62)	4 (17)	
negative	5 (38)	20 (83)	p = 0.005 (chi-2)

### Correlation of LOH at 19q to clinical outcome in the study

**Figure 3:**
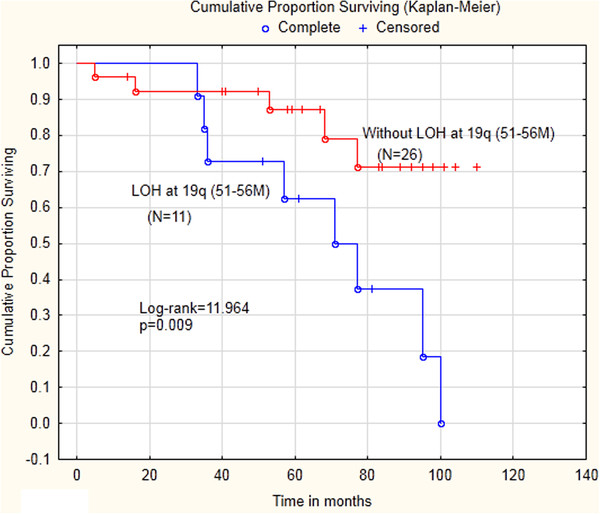
**Patients with LOH at chromosome 19q (51-56 Mbp) are associated with shorter survival.** Kaplan-Meier plot indicates significant differences in overall survival between patientis with and without LOH on chromosome 19q, Log-rank test =12.0; (p < 0.009).

Recurrent disease was related to FIGO- stage (Table 2), but not to tumor grade or type of staging at primary surgery. However, FIGO-stage was not an independent prognostic factor in this series of patients. Tumor grade (G1 + G2 *vs.* G3) was significantly (p = 0.02) related to FIGO-stage (IA-IC *vs.* IIA-IIC). Only two out of the 15 high grade tumors (G3) belonged to FIGO-stage IA. In a survival analysis (Figure 3), LOH at 19q (51-56 Mbp) was significantly correlated with shorter disease-free survival (DFS) (Log-rank = 12.0; p = 0.009). In order to study the effect of LOH in relation to other factors we performed a multivariate Cox regression analysis with DFS as endpoint including other factors such as age, stage, grade and staging at primary surgery (Table 3). As the clinical outcome of the moderate-grade carcinomas is more similar to that of low-grade carcinomas, we chose to compare low and moderate grades with the high grade carcinomas in the multivariate analysis [25]. The results show that LOH at 19q (51-56Mbp) was an independent prognostic factor with HR = 4.5 (95% CI = 1.07-18.6) and no other factor was significant In a separate multivariate Cox regression analysis with DFS as endpoint (Table 4), no statistically significant association was found with TP53 status (p = 0.077). Furthermore, LOH at 19q (51-56Mbp) also was an independent predictive factor for recurrent disease (Table 5) with OR = 19.0 (95% CI = 1.39- 261) in a logistic regression analysis with the same variables as before (age, stage, grade and staging at primary surgery).

**Table 3:** **Multivariate Cox regression analysis with disease-free survival as endpoint** (N = 37)

**Variable**	**HR**	**95% CI**	**P value**
Age	1.06	0.988-1.13	0.105
Stage (I vs. II)	2.65	0.532-13.2	0.234
Grade*	1.26	0.2386.64	0.786
Staging**	0.527	0.057-4.85	0.572
LOH 19q***	4.47	1.071-18.6	0.039

* G1 or G2 vs. G3.

** Staging surgery adequately performed vs. not performed.

*** LOH at 19q detected vs. not detected.

*HR* Hazard Rate.

**Table 4:** **Multivariate Cox regression analysis with disease-free survival as endpoint** (N = 37)

**Variable**	**HR**	**95% CI**	**P value**
Age	1.05	0.988-1.13	0.138
Stage (I vs. II)	3.98	0.939-16.9	0.060
Grade *	0.746	0.185-3.00	0.680
Staging **	0.492	0.050-4.78	0.541
TP53 status ***	2.92	0.888-9.64	0.077

* G1 or G2 vs. G3.

** Staging surgery adequately performed vs. not performed.

*** TP53 immunohistological staining detected vs. not detected.

HR = Hazard Rate.

**Table 5:** **Logistic regression analysis with recurrent disease as endpoint** (N = 37)

**Variable**	**OR**	**95% CI**	**P value**
Age	1.06	0.148-820	0.264
Stage (I vs. II)	15.3	0.436-537	0.127
Grade *	0.313	0.024-4.06	0.363
Staging **	0.197	0.007-5.34	0.323
LOH 19q***	19.0	1.39-261	0.028

* G1 or G2 vs. G3.

** Staging surgery adequately performed vs. not performed.

*** LOH at 19q detected vs. not detected.

*OR* Odds Ratio.

## Discussion

In epithelial ovarian cancer, which is a heterogeneous and cytogenetically complex disease, DNA-microarray technology gene-expression profiling has been used to provide prognostic information, to predict response to first-line platinum-based chemotherapy, and to discriminate between different histological subtypes [26]. New Bioinformatic tools such as TAPS make it possible to analyse allele-specific copy numbers and LOH in the tumor cells [16]. The OncoScan technology featuring molecular inversion probes facilitated the analysis of FFPE samples, thereby enabling access to large collections of archival samples. Our analysis of FFPE samples using conventional Affymetrix 250K arrays had a lower success rate (20%) compared to 73% using OncoScan, probably due to different DNA quality requirements.

In the present study, the FIGO-stage was significant for recurrent disease in univariate analysis but not an independent factor in multivariate analysis. However, both FIGO stage and TP53 status were independent prognostic and predictive factors in multivariate analysis in previous studies [18-20]. Tumor grade was not an independent factor in multivariate analysis in any of the previous studies. One difference between the studies is that previous studies have assessed tumors of five different histo-pathological subtypes of EOC, while the present study is restricted to only serous tumors. However, an important explanation of the different findings in multivariate analyses is the limited number of 37 cases in the present work that may not have provided sufficient power to detect significance in other variables.

The 37 ovarian cancer samples were divided into two subgroups those from patients with or without recurrent disease. Significant differences in aberration frequencies were detected for amplification (gain), deletion (loss) and LOH. Our findings of recurrent gain (8p, 9p, 11q and 17q) and loss (2p, 4q, 5q, 8q, 9q, 10q 15q, 17p, 17q, 19p and 21q) were in agreement with other studies on ovarian cancer in all stages [27-30]. This indicates that for this disease, the early-stage alteration pattern resembles that of the disease in general, and that adjusting the copy number analysis for aberrant average ploidy does not significantly alter the regions with observed recurrent gain and loss. The allele-specific analysis of copy numbers in this study also provides information on LOH. LOH is of particular importance since it enables recessive mutations (in tumor suppressor genes) to influence the tumor phenotype. While copy number or LOH analysis does not identify point mutations directly, regions with high frequency of LOH are candidates for harboring mutations in tumor suppressor genes [31]. We found a high frequency of LOH (up to 80%) around TP53 on 17p, a gene known to be mutated in different kinds of cancer [32]. LOH at 17p is common in serous ovarian cancer with a frequency of 65% in one study [32] and 71% in another [33]. In a study from The Cancer Genome Atlas (TCGA) TP53 was found to be mutated in 303 out of 316 (96%) high-grade serous ovarian cancer (HGPSC) [29]. The high prevalence of mutation in high-stage cases suggested that is an important event in the pathogenesis of HGPSC. From the validation set in one study [34], 15 cases were stage I or II HGPSC and 13/15 (86.7%) had TP53 mutations.

In addition, we observed that LOH was particularly frequent on chromosome 4q, implying that a tumor suppressor gene may reside in this part of the genome. In contrast to 17p no tumor suppressor gene has been identified on chromosome 4q. Furthermore, regions of frequent LOH (>36%) were located on chromosome 16 in our study. In a study from Gorringe et al [31] expression of genes in regions of frequent LOH (>35%) were most frequently found on chromosome 17 and chromosome 19. When differences in LOH between the groups were analysed in our study, regions of relatively limited size on chromosome 19 had particularly large differences in frequency of LOH (> 36%). There was no overlap with the regions presented by Gorringe et al, which is likely to be explained by differences in ascertaining the cohorts.

Another important finding in our study was that presence of LOH in segments at 19q (51-56Mbp) was significantly associated/correlated with clinical outcome. In survival analysis patients with presence of LOH in segments at 19q was identified to have poor prognosis concerning survival. Furthermore, in a multivariate analysis with DFS and recurrent disease as endpoints presence of LOH in segments at 19q (51-56Mbp) of early-stage serous ovarian cancer were shown to be significant independent prognostic and predictive factors, These results are in line with another study on ovarian cancer where twenty patients were included and LOH on chromosome 19q 13.2-13.4 [35] in 53% of cases was noted. It can also be noted that TP53 status assessed by immunohistochemistry was not significant as an independent prognostic marker in contrast to LOH 19q in this study (Tables 3 and 4). It is well known that TP53 is frequently mutated in high grade serous cancers [29]. However in this study only 15/37 (41%) of tumors were classified as high grade. Thus, the low frequency of high grade tumors in this study may explain the lack of significance.

From regions where the most significant differences (p < 0.01) in LOH were seen on chromosome 19q, a total of 56 genes could be identified, that were potentially candidate genes associated with clinical outcome. Nine of the 56 genes in the region on 19q were described to be expressed in ovarian carcinoma in The Human Protein Atlas [36] (MARCH2, ANGPTL4, HNRNPM, ADAMTS10, NAPA, SLC1A5, CCDC8, CCDC9, BBC3 (PUMA)) and should receive extra attention when trying to identify candidate genes for harboring tumor suppressor mutations that could be associated with clinical outcome in further studies.

Positive immunostaining of TP53 does not provide direct information on the *TP53* mutation status. For instance null mutations can be expected to be undetected, especially if no protein is made. Thus, it is interesting that, in spite of the limitations of immunostaing, we find a significant association between TP53-positivity and LOH at 19q (51-56Mbp). It is known from other tumor studies for instance in chronic lymphocytic leukaemia that there may be a significant co-occurrence of different aberrations [37]. One hypothesis is that the aberrations in the two regions co-operate in causing a tumor phenotype that leads to recurrent disease. In this context it is intriguing that one of the genes located on 19q (51-56 Mbp), *BBC3,* is normally expressed in ovarian cancer and has been shown to be regulated by TP53 and is required for TP53-dependent apoptosis in a previous study [38]. Recent results also show that patients with concomitant low expression of BBC3 *(PUMA)*, and mutated TP53 in the tumor cells have worse survival than others [18]. A strong correlation has been observed between a TP53 mutation, and the expression of the TP53 protein, identified by the monoclonal antibody DO-7 [11,39]. Taken together these results suggest that a lower expression level of BBC3 may contribute to the phenotype. In addition loss-of-function mutations in *BBC3* could also contribute to the phenotype. One study in head and neck and lung cancer have identified LOH on 19q but no mutations could be found in coding regions of *BBC3* in 30 tumors [40]. Studies of somatic mutations in genes on 19q and in *BBC3* in particular would be an interesting topic for further studies on the mechanisms underlying recurrence and poor outcome in early-stage serous ovarian cancer.

## Conclusions

Recent advances in microarray technology and data analysis has facilitated the study of this patient cohort on 37 early-stage patients with high-grade and low/medium grade serous ovarian tumors. The study has identified a number of genomic regions where copy number aberrations are linked to recurrent disease in early stage serous ovarian cancers. In particular LOH on chromosome 19q is associated with TP53 mutation and poor clinical outcome. Thus, further studies on identifying the molecular mechanism underlying these results are motivated and will hopefully improve our understanding on factors determining prognosis for patients.

## Competing interests

The authors declare no competing interests.

## Authors' contributions

IS: Collection and characterization of patient material, statistical analysis. Formulation of research questions and interpretation of results. MM: Data analysis and interpretation of results. MR: DNA handling and array analysis. HÅ: Interpretation of results. AI: formulation of research questions, supervision of data analysis and interpretation of results. IS, MM, HÅ and AI wrote the paper. All authors read and approved the final manuscript.

## Pre-publication history

The pre-publication history for this paper can be accessed here:

http://www.biomedcentral.com/1471-2407/12/407/prepub

## Supplementary Material

Additional file 1 Frequencies of gain, loss and LOH in samples with and without recurrence. Click here for file

Additional file 2 Regions of significant difference in gain, loss and LOH for samples with (group 1) and without (group 2) recurrence. Click here for file
