# Supplementary material for: Loss-of-heterozygosity on chromosome 19q in early-stage serous ovarian cancer is associated with recurrent disease
Source: BMC Cancer. 2012 Sep 12;12:407. doi: 10.1186/1471-2407-12-407 (PMC3495882; doi:10.1186/1471-2407-12-407)
Supplement: Additional file 1 — Frequencies of gain, loss and LOH in samples with and without recurrence. [file 1471-2407-12-407-S1.pdf]

Supplement 1.
Frequencies of gain, loss and LOH in samples with and without recurrence

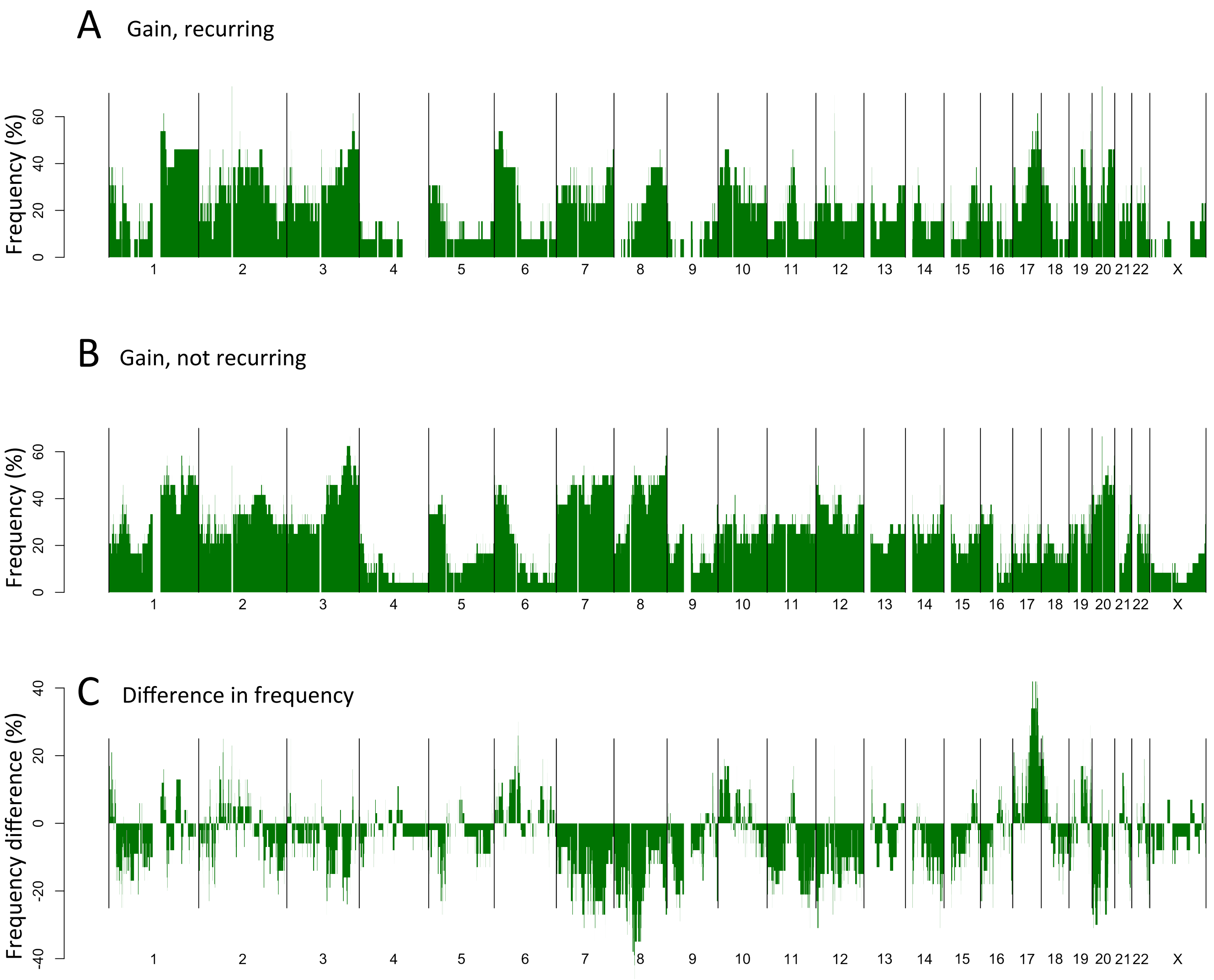
A Gain, recurring
B Gain, not recurring
C Difference in frequency
Frequency (%)
Frequency difference (%)
0
20
40
60
-20
-40
1
2
3
4
5
6
7
8
9
10
11
12
13
14
15
16
17
18
19
20
21
22
X

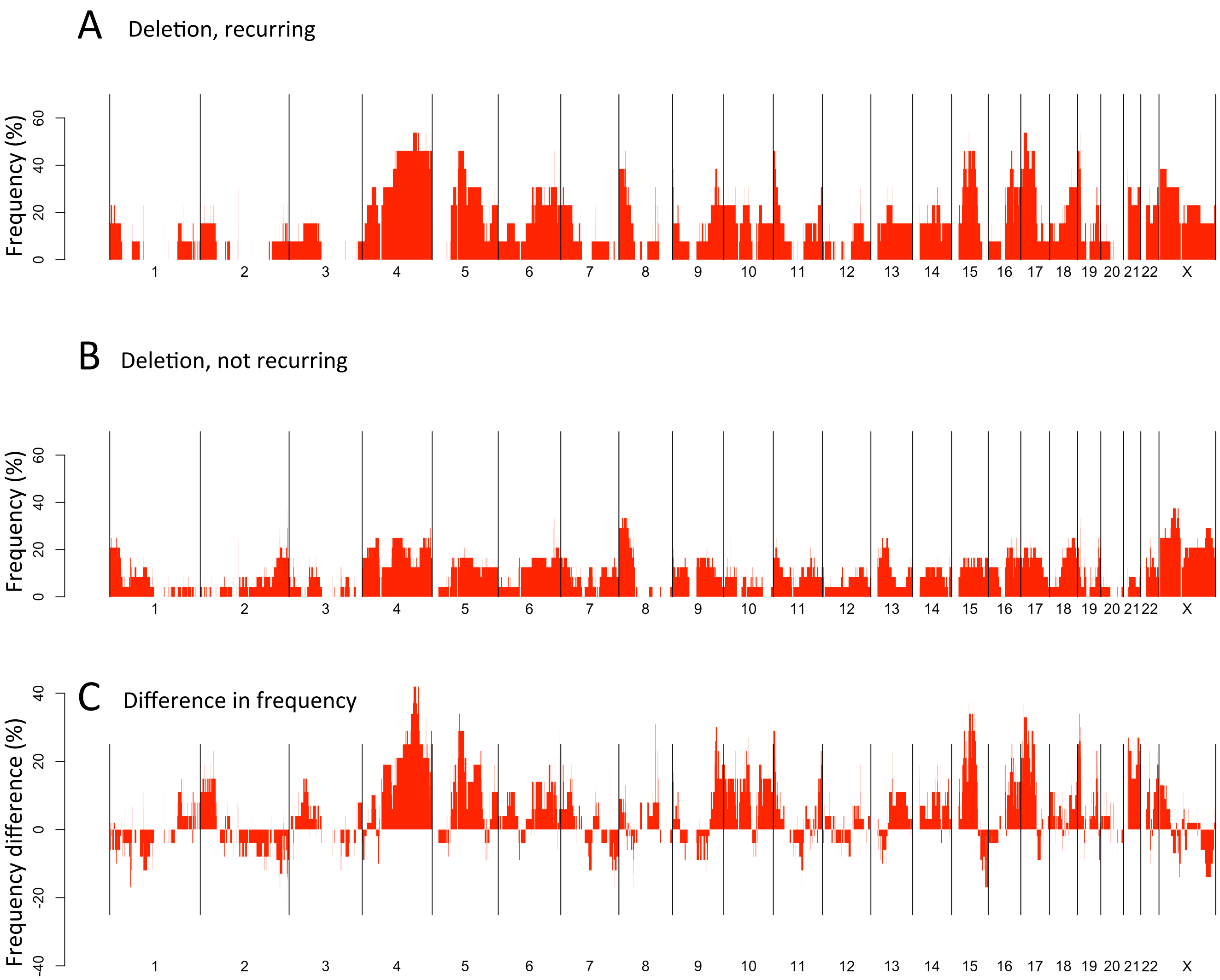
A Deletion, recurring
Frequency (%)
0
20
40
60
1 2 3 4 5 6 7 8 9 10 11 12 13 14 15 16 17 18 19 20 21 22 X
B Deletion, not recurring
Frequency (%)
0
20
40
60
1 2 3 4 5 6 7 8 9 10 11 12 13 14 15 16 17 18 19 20 21 22 X
C Difference in frequency
Frequency difference (%)
-40
-20
0
20
40
1 2 3 4 5 6 7 8 9 10 11 12 13 14 15 16 17 18 19 20 21 22 X

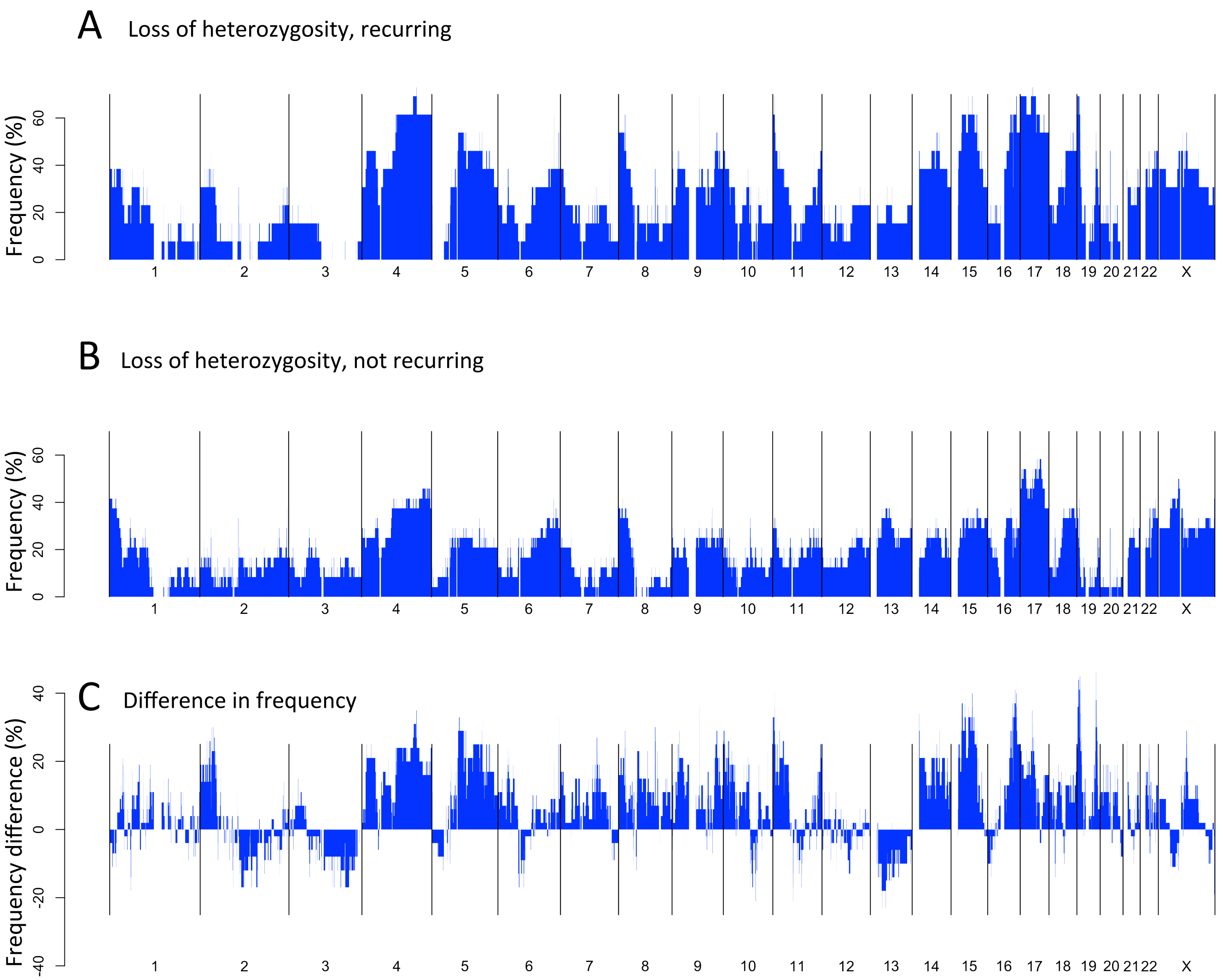
A
Loss of heterozygosity, recurring
Frequency (%)
0
20
40
60
1 2 3 4 5 6 7 8 9 10 11 12 13 14 15 16 17 18 19 20 21 22 X
B
Loss of heterozygosity, not recurring
Frequency (%)
0
20
40
60
1 2 3 4 5 6 7 8 9 10 11 12 13 14 15 16 17 18 19 20 21 22 X
C
Difference in frequency
Frequency difference (%)
-40
-20
0
20
40
1 2 3 4 5 6 7 8 9 10 11 12 13 14 15 16 17 18 19 20 21 22 X
