# Supplementary material for: Loss-of-heterozygosity on chromosome 19q in early-stage serous ovarian cancer is associated with recurrent disease
Source: BMC Cancer. 2012 Sep 12;12:407. doi: 10.1186/1471-2407-12-407 (PMC3495882; doi:10.1186/1471-2407-12-407)
Supplement: Additional file 2 — Regions of significant difference in gain, loss and LOH for samples with (group 1) and without (group 2) recurrence. [file 1471-2407-12-407-S2.pdf]

Supplement 2.
Regions of significant difference in gain, loss and and LOH for samples with (group 1) and without (group 2) recurrence.

Gain

Chromosome	Start	End	Count1	Count2	percent1	percent2	percent dif	p value
chr8	56 861 697	57 029 201	1	13	8	54	-46	1,11%
chr8	57 029 201	57 166 510	1	13	8	54	-46	1,11%
chr8	56 083 107	56 861 697	1	12	8	50	-42	1,29%
chr17	53 418 671	53 706 750	7	3	54	12	42	1,67%
chr17	53 706 750	56 304 372	7	3	54	12	42	1,67%
chr17	61 750 932	62 150 619	7	3	54	12	42	1,67%
chr17	62 505 533	64 149 283	7	3	54	12	42	1,67%
chr17	65 784 017	66 131 953	7	3	54	12	42	1,67%
chr17	66 131 953	66 159 746	7	3	54	12	42	1,67%
chr8	50 792 956	51 638 549	1	11	8	46	-38	2,72%
chr8	51 638 549	51 788 906	1	11	8	46	-38	2,72%
chr8	51 788 906	53 267 926	1	11	8	46	-38	2,72%
chr8	53 267 926	53 729 833	1	11	8	46	-38	2,72%
chr17	62 150 619	62 203 925	7	4	54	17	37	2,75%
chr17	62 476 999	62 505 533	7	4	54	17	37	2,75%
chr17	66 159 746	66 913 192	7	4	54	17	37	2,75%
chr17	67 095 036	67 463 601	8	5	62	21	41	2,82%
chr17	67 463 601	67 519 088	8	5	62	21	41	2,82%
chr17	67 519 088	67 547 371	8	5	62	21	41	2,82%
chr17	67 547 371	67 553 395	8	5	62	21	41	2,82%
chr8	36 249 824	37 004 390	0	8	0	33	-33	3,24%
chr8	55 276 747	55 601 143	2	13	15	54	-39	3,53%
chr8	57 166 510	57 921 033	2	13	15	54	-39	3,53%
chr11	113 393 056	113 399 897	0	7	0	29	-29	3,78%
chr11	113 399 897	113 445 150	0	7	0	29	-29	3,78%
chr8	34 122 993	36 249 824	0	7	0	29	-29	3,78%
chr9	35 333 754	35 719 749	0	7	0	29	-29	3,78%
chr9	35 719 749	36 216 273	0	7	0	29	-29	3,78%
chr17	67 553 395	67 578 174	8	6	62	25	37	3,95%
chr17	68 153 023	69 884 739	8	6	62	25	37	3,95%
chr17	69 884 739	70 096 026	8	6	62	25	37	3,95%
chr17	50 297 462	53 418 671	6	3	46	12	34	4,25%
chr17	56 304 372	56 361 263	6	3	46	12	34	4,25%
chr17	56 361 263	60 827 118	6	3	46	12	34	4,25%
chr17	60 827 118	61 265 344	6	3	46	12	34	4,25%
chr17	61 265 344	61 750 932	6	3	46	12	34	4,25%
chr17	64 149 283	64 444 897	6	3	46	12	34	4,25%
chr17	65 047 636	65 784 017	6	3	46	12	34	4,25%

Loss

Chromosome	Start	End	Count1	Count2	percent1	percent2	percent dif	p value
chr8	99 457 577	101 247 298	4	0	31	0	31	1,08%
chr10	120 943 502	121 198 451	4	0	31	0	31	1,08%
chr15	57 994 767	58 064 025	6	2	46	8	38	1,34%
chr15	61 362 436	61 549 691	6	2	46	8	38	1,34%
chr4	139 989 043	140 114 371	7	3	54	12	42	1,67%
chr4	142 273 477	147 896 176	7	3	54	12	42	1,67%
chr4	152 820 372	154 877 563	7	3	54	12	42	1,67%
chr4	140 114 371	142 273 477	7	4	54	17	37	2,75%
chr4	147 896 176	149 213 648	7	4	54	17	37	2,75%
chr4	149 630 049	149 845 502	7	4	54	17	37	2,75%
chr15	47 062 713	47 945 433	7	4	54	17	37	2,75%
chr17	7 903 881	9 559 568	7	4	54	17	37	2,75%
chr19	7 078 334	7 084 047	7	4	54	17	37	2,75%
chr9	74 704 525	74 891 278	8	5	62	21	41	2,82%
chr2	35 668 134	35 943 775	3	0	23	0	23	3,68%
chr2	36 484 725	36 675 131	3	0	23	0	23	3,68%
chr8	99 394 050	99 457 577	3	0	23	0	23	3,68%
chr8	101 247 298	101 399 057	3	0	23	0	23	3,68%
chr8	106 197 508	107 134 620	3	0	23	0	23	3,68%
chr10	61 456 386	63 684 032	3	0	23	0	23	3,68%
chr10	111 929 832	112 054 945	3	0	23	0	23	3,68%
chr10	120 935 739	120 943 502	3	0	23	0	23	3,68%
chr10	121 198 451	121 207 101	3	0	23	0	23	3,68%
chr10	67 901 535	68 174 223	4	1	31	4	27	4,23%
chr21	12 300 000	15 024 609	4	1	31	4	27	4,23%
chr21	33 709 869	34 118 458	4	1	31	4	27	4,23%
chr21	37 545 381	38 099 884	4	1	31	4	27	4,23%
chr21	38 205 310	42 909 904	4	1	31	4	27	4,23%
chr4	132 850 694	139 989 043	6	3	46	12	34	4,25%
chr4	149 981 374	152 820 372	6	3	46	12	34	4,25%
chr4	154 877 563	156 493 522	6	3	46	12	34	4,25%
chr5	73 836 322	76 354 453	6	3	46	12	34	4,25%
chr15	48 141 732	53 625 528	6	3	46	12	34	4,25%
chr15	56 149 154	57 994 767	6	3	46	12	34	4,25%
chr15	58 064 025	58 535 612	6	3	46	12	34	4,25%
chr15	60 842 591	61 362 436	6	3	46	12	34	4,25%
chr15	61 549 691	61 773 888	6	3	46	12	34	4,25%
chr15	64 226 967	64 885 808	6	3	46	12	34	4,25%
chr17	33 120 567	33 285 409	6	3	46	12	34	4,25%
chr19	4 458 118	4 719 760	6	3	46	12	34	4,25%
chr19	4 917 027	5 692 639	6	3	46	12	34	4,25%

LOH

Chromosome	Start	End	Count1	Count2	percent1	percent2	percent dif	p value
chr19	51 677 948	53 020 004	7	2	54	8	46	0,41%
chr19	8 032 100	8 613 664	8	4	62	17	45	0,97%
chr19	8 613 664	8 810 515	8	4	62	17	45	0,97%
chr19	51 587 851	51 677 948	6	2	46	8	38	1,34%
chr19	53 020 004	53 221 395	6	2	46	8	38	1,34%
chr16	79 057 628	79 130 500	9	6	69	25	44	1,45%
chr19	4 458 118	4 719 760	9	6	69	25	44	1,45%
chr19	4 719 760	4 917 027	9	6	69	25	44	1,45%
chr19	4 917 027	5 692 639	9	6	69	25	44	1,45%
chr19	7 078 334	7 084 047	9	6	69	25	44	1,45%
chr19	8 921 313	8 928 556	7	3	54	12	42	1,67%
chr19	8 810 515	8 921 313	7	4	54	17	37	2,75%
chr16	74 054 227	74 613 264	8	5	62	21	41	2,82%
chr16	76 655 105	77 239 446	8	5	62	21	41	2,82%
chr19	6 028 925	6 841 327	8	5	62	21	41	2,82%
chr19	6 841 327	6 898 422	8	5	62	21	41	2,82%
chr19	7 620 258	8 032 100	8	5	62	21	41	2,82%
chr9	74 699 163	74 701 284	9	7	69	29	40	3,57%
chr9	74 701 284	74 704 525	9	7	69	29	40	3,57%
chr9	74 704 525	74 705 217	9	7	69	29	40	3,57%
chr11	4 377 654	4 531 730	9	7	69	29	40	3,57%
chr15	57 284 534	57 994 767	9	7	69	29	40	3,57%
chr15	57 994 767	58 064 025	9	7	69	29	40	3,57%
chr16	77 601 931	77 722 783	9	7	69	29	40	3,57%
chr16	77 722 783	78 061 961	9	7	69	29	40	3,57%
chr16	78 061 961	78 109 771	9	7	69	29	40	3,57%
chr16	78 709 798	78 994 103	9	7	69	29	40	3,57%
chr16	79 033 438	79 057 628	9	7	69	29	40	3,57%
chr7	101 761 990	102 122 842	3	0	23	0	23	3,68%
chr8	50 792 956	57 166 510	3	0	23	0	23	3,68%
chr8	79 612 514	79 848 270	3	0	23	0	23	3,68%
chr15	29 423 998	31 387 439	8	6	62	25	37	3,95%
chr15	31 387 439	31 453 251	8	6	62	25	37	3,95%
chr15	38 116 096	38 880 320	8	6	62	25	37	3,95%
chr16	68 261 825	69 183 893	8	6	62	25	37	3,95%
chr16	69 825 512	70 254 082	8	6	62	25	37	3,95%
chr16	73 486 905	74 054 227	8	6	62	25	37	3,95%
chr16	74 613 264	76 655 105	8	6	62	25	37	3,95%
chr16	77 239 446	77 253 638	8	6	62	25	37	3,95%
chr16	79 130 500	79 151 618	8	6	62	25	37	3,95%
chr19	5 692 639	6 028 925	8	6	62	25	37	3,95%
chr19	6 898 422	7 012 134	8	6	62	25	37	3,95%
chr19	7 012 134	7 060 449	8	6	62	25	37	3,95%
chr19	7 060 449	7 078 334	8	6	62	25	37	3,95%
chr2	38 322 587	39 428 906	4	1	31	4	27	4,23%
chr7	99 051 354	99 242 585	4	1	31	4	27	4,23%
chr7	99 283 903	99 842 356	4	1	31	4	27	4,23%
chr19	12 748 742	13 016 909	4	1	31	4	27	4,23%
chr14	18 675 413	19 476 620	6	3	46	12	34	4,25%
chr19	8 928 556	9 440 779	6	3	46	12	34	4,25%
chr19	9 440 779	9 561 234	6	3	46	12	34	4,25%
chr19	55 140 224	55 371 999	6	3	46	12	34	4,25%
chr9	74 705 217	74 891 278	9	8	69	33	36	4,70%
chr11	4 531 730	4 804 600	9	8	69	33	36	4,70%
chr15	47 062 713	47 945 433	9	8	69	33	36	4,70%
chr15	58 064 025	58 528 636	9	8	69	33	36	4,70%
chr16	78 109 771	78 410 845	9	8	69	33	36	4,70%
chr16	78 410 845	78 709 798	9	8	69	33	36	4,70%
chr16	78 994 103	79 033 438	9	8	69	33	36	4,70%
chr19	1 739 970	4 458 118	9	8	69	33	36	4,70%
chr19	7 084 047	7 093 939	9	8	69	33	36	4,70%
